# Resourcing research on social semantic deficits in dementia: Psycholinguistic norms for Spanish‐speaking cohorts

**DOI:** 10.1002/alz70856_099136

**Published:** 2025-12-25

**Authors:** Pamela Lopes da Cunha, Agustina Selser, Lucia Amoruso, Adolfo M. Garcia, Ivan Caro

**Affiliations:** ^1^ Consejo Nacional de Investigaciones Científicas y Técnicas (CONICET), Ciudad Autónoma de Buenos Aires, Buenos Aires, Argentina; ^2^ Cognitive Neuroscience Centre, University of San Andres, Victoria, Buenos Aires, Argentina; ^3^ Basque Center on Cognition, Brain, and Language, San Sebastian, Gipuzkoa, Spain; ^4^ Universidad Santiago de Chile, Estación Central, Santiago de Chile, Chile; ^5^ Global Brain Health Institute, University of California, San Francisco, CA, USA; ^6^ Consejo Nacional de Investigaciones Científicas y Técnicas (CONICET), Buenos Aires, Argentina

## Abstract

**Background:**

Emerging research suggests that behavioral‐variant frontotemporal dementia (bvFTD) involves distinct deficits in processing social concepts –units that denote interpersonal traits, events, and circumstances. Recent findings indicate that assessments of these domains could contribute to differential diagnosis and predict syndrome‐specific neural alterations. However, unlike other semantic categories, social concepts lack normative datasets for under‐represented languages, which hinders strategic stimulus selection for much‐needed experiments on under‐served populations. To tackle this gap, we created a normative psycholinguistic database of social and non‐social concepts for Spanish‐speaking Latinos, aimed to fuel groundbreaking research on this population.

**Methods:**

Healthy subjects over 18 years old completed an online form, rating 600 Spanish words (nouns, verbs, adjectives) in terms of their meanings’ social relevance. Subsets of 100 words with high and low sociality (e.g., “friendship” and “button”, respectively) were randomly presented. After rating their comprehension of the instructions, participants assessed the sociality of each word using a Likert scale (1 = no sociality, 7 = high sociality). Stimuli selection was made based on Diveica et al. (Behav Res Methods 2023, 54:461‐73), who achieved strong validity in categorizing social and non‐social English words, and socialness‐driven variance in lexical tasks. Participants were recruited through flyers in online educational platforms and social media.

**Result:**

Socialness ratings averaged 5.997 (*SD* = 0.873) for social words and 1.75 (*SD* = 0.909) for non‐social words. Participants provided more consistent responses at the extremes of the scale. These findings suggest strong face validity and a clear distinction between categories.

**Conclusions:**

Focusing on linguistic tools, we aim for a comprehensive study of social concepts and their potential as markers of cerebral dysfunction. In particular, we hope to enhance clinical investigation and diagnoses for populations with bvFTD, while fostering a fertile ground for novel translational research. This Spanish database is crucial for data collection in underrepresented populations and contributes to equity in dementia research and, more broadly, in behavioral neurology, by incorporating the Latin American perspective.

